# The use of health apps in primary care—results from a survey amongst general practitioners in Germany

**DOI:** 10.1007/s10354-021-00814-0

**Published:** 2021-02-11

**Authors:** Julian Wangler, Michael Jansky

**Affiliations:** Research Associate | Centre for General Medicine and Geriatrics, Universitätsmedizin Mainz, Am Pulverturm 13, 55131 Mainz, Germany

**Keywords:** Health apps, mHealth, Prevention, Health promotion, Primary healthcare, Gesundheits-Apps, mHealth, Prävention, Gesundheitsförderung, Hausarzt

## Abstract

Mass availability and use of health apps raises the question as to how they might be integrated into healthcare systems towards improving prevention and therapy. This study has researched prevailing opinion on health apps amongst primary care physicians, potential application areas physicians have seen in their experience with these apps up to now, and situations suitable for using apps in patient care. A total of 2138 primary care physicians in the state of Baden-Württemberg, Germany, responded to an anonymised written survey between March and June 2020. Physicians with a positive opinion (36%) emphasised motivation and compliance as advantages, whereas sceptical respondents (43%) expressed suspicion regarding data privacy and reliability as well as legal issues and additional workload arising from using the apps. Even so, a clear majority accepted the potential benefit from sensible use of health apps with features providing prevention and lifestyle support (90/76%). With respect to patients using the apps, 54% of respondents saw a positive contribution to healthcare and/or recovery. Despite the perceived benefits of health apps, general practitioners are still reluctant to bring up or recommend health apps in their consultations. Many physicians do not feel capable of giving expert advice to patients on the apps available. Many general practitioners are aware of the potential that health apps may have in improving prevention and treatment. However, there are reservations and uncertainties regarding clarity, transparency, and privacy issues in these apps. More focus should be placed on these concerns to ensure ideal conditions for integrating health apps into primary care.

## Abstract (de)

Mit der zunehmendem Durchsetzung und Verwendung von Gesundheits-Apps durch Verbraucher stellt sich die Frage nach deren Integration ins Gesundheitswesen, sodass ein Nutzen für Prävention und Therapie erzielt werden kann. Die Studie untersucht die Einstellungen von Hausärzten in Bezug auf Gesundheits-Apps, inwiefern diese aus ihrer Erfahrung Einsatzpotenziale wahrnehmen und unter welchen Bedingungen Apps stärker in die Primärversorgung einbezogen werden können. Zwischen März und Juni 2020 wurden insgesamt 2138 Hausärzte in Baden-Württemberg anonymisiert befragt. Ärzte mit aufgeschlossener Haltung sehen in Bezug auf Gesundheits-Apps Motivations- und Compliance-Vorteile (36 %), während skeptische Ärzte Aspekte wie Datensicherheit, Verlässlichkeit, rechtliche Sicherheiten bei der Anwendung und mögliche Mehrbelastungen im Blick haben (43 %). Dennoch nehmen die meisten Befragten klare Mehrwerte und Anwendungspotenziale von Apps wahr, insbesondere im Hinblick auf Präventions- und den Lebensstil unterstützende Funktionen (90/76 %). Positive Effekte von Apps bei der Gesundheitsvorsorge bzw. Genesung haben bereits 54 % der Hausärzte beobachtet. Trotz wahrgenommener Vorzüge von Gesundheits-Apps sind Hausärzte bei der aktiven Thematisierung und/oder Empfehlung solcher Programme eher zurückhaltend. Ein Großteil der Ärzte traut sich nicht zu, Patienten kompetent in Bezug auf verfügbare Apps zu beraten. Resümierend lässt sich festhalten, dass viele Hausärzte in Gesundheits-Apps positives Potenzial für Präventions- und therapeutische Maßnahmen wahrnehmen. Allerdings sollten bestehende Vorbehalte und Unsicherheiten in Bezug auf Überschaubarkeit, Transparenz und Anwendung verstärkt adressiert werden, um die richtigen Voraussetzungen für eine erfolgreiche Integration in die primärärztliche Versorgung zu schaffen.

## Introduction

Studies have demonstrated that about 50% of smartphone users frequently or regularly use a health app [1–3]. These mHealth applications are aimed at disease prevention, monitoring or therapy [4]. Currently, health apps are mainly used for recording physical and health data as well as providing information on health and nutrition issues, monitoring symptoms and supporting patients in their management of medicines [5, 6].

Health apps have been associated with patient empowerment by potentially contributing to instilling healthy behaviour and encouraging therapy through a combination of low-level application and continuous motivation as well as reminder features [7, 8]. They may also conceivably help in recognising health risks earlier while boosting the effectiveness of doctor–patient relationships [9]. Health apps are designed for easy integration in everyday life [1, 3, 4].

Criticism of health apps often focuses on the lack of data privacy as well as unclear policies on what happens to the collected data [1]. Some have warned that health apps could produce measurement errors, or that incorrect application could lead to erroneous diagnosis and treatment [5]. Others also expressed concerns that what are referred to as *symptom checkers* may also encourage patients to self-diagnose and self-treat, possibly also leading to health anxiety [10].

Health apps have so far not been subjected to any administrative licencing or other regulations [11]. The German federal parliament passed a law in November 2019 making it easier in the long term for health insurers to cover the costs of health apps as medical products after detailed examination [12].

Studies on the actual effectiveness of these mHealth tools have so far only been sporadic [4, 13]. As an example, there are only a few usage studies in postoperative patient follow-up [14, 15]. Even so, the CHARISMHA metastudy held that health apps have shown success at the boundary between a lifestyle product and treatment programme, such as in monitoring patients with diabetes mellitus type 2 [1].

The apps may be of particular benefit in primary care considering the heavy pressure on time and resources as well as the wide range of symptoms, diseases and patient groups involved [16]. The apps would seem ideal as support tools for physicians to use on specific patients in primary care, thus promoting health in the long term [17, 18]. Studies have also emphasised the benefits of apps as an aid in optimising differential diagnosis as well as patient and treatment compliance [8, 9].

Only a few studies have examined the application potential for health apps specifically in primary care [11, 19, 20]. Surveys amongst mixed specialist groups have shown that 25–45% of physicians occasionally discuss health apps with their patients [16, 19, 21]. Apart from that, 36–42% see reinforced patient empowerment as a major benefit in these apps, followed by improved patient training. Surveys conducted in the US and Australia indicate relative reluctance amongst medical professionals in raising the topic of digital or mobile health monitoring solutions with their patients [22, 23]. Physicians may see opportunities for health apps in reinforcing patient motivation and empowerment [24], but express uncertainty as to whether the apps are ready for prime time in view of reliability, data privacy, technical maturity and applicability, and their everyday implementation in patient care systems [18, 25].

The aim of the present study was to investigate the extent to which health apps may provide benefit or useful support in patient care from a general practitioner’s point of view. To this end, this study sets out to address the following questions:

- What is the general opinion amongst general practitioners regarding health apps? Where do they see opportunities, and where do they see threats?
- What experiences have general practitioners had with health apps with respect to patient care? How extensively are they actively used?
- Under what conditions could the potential in health apps be more effectively utilised in patient care, specifically primary care?

## Materials and methods

### Study design and setting

An exploratory approach seems appropriate given the lack of empirical knowledge in this field. A qualitative preliminary study was carried out beforehand with 35 general practitioners interviewed on potential application and support from health apps in primary care [26]. After that, a written, anonymous survey of primary care physicians was conducted in the German federal state of Baden-Württemberg between March and June 2020. This study aimed at gauging attitudes, experiences and opinions amongst primary care physicians with respect to health apps.

### Recruitment and participants

All 6760 general practitioners currently active as primary care physicians in Baden-Württemberg were invited to participate in the anonymous survey. Responding general practitioners were not given any remuneration for participating. The questionnaires were collected by the authors and then evaluated by the team.

### Questionnaire and sociodemographic variables

The questionnaire was developed along the central categories of the guidelines for the qualitative interview study [26]. Based on the results of this preliminary study, it has been shown that general practitioners assess health apps from very different perspectives. Physicians with a positive attitude argue, for example, with motivational and compliance advantages and a gamification approach of many apps, while sceptical physicians articulate a mistrust in terms of data security, functionality and legal issues when including apps. For this reason, it was important for the development of the questionnaire to reflect the spectrum of opinions represented by the interview study as broadly as possible.

In addition, a literature search was carried out. In the course of this, studies relevant to the research topic were incorporated into the construction of the questionnaire. These included in particular the CHARISMHA metastudy, with the help of which different fields of application for apps were systematized but also optimization approaches were compiled [1, 4]. The work by Dufour et al. [27] and Ernsting et al. [28, 29] served to develop questions that were formulated as closely as possible to the reality of general practitioner care.

The final questionnaire covered four topical blocks:

- Acceptance of health apps; opportunities and threats in various applications
- Importance and use of health apps in patient care according to personal experience
- Information behaviour and competence assessment with respect to health apps
- Potential for improvement towards increasing the attractiveness of health apps in primary care

Sociodemographic characteristics such as age, gender, doctor’s office setting, type of doctor’s office and patients were collected for each quarter.

### Pretest

A pretest was carried out before the actual survey. The questionnaire was presented to a total of 15 general practitioners in order to check the comprehensibility and completeness of the categories. It was also checked whether the questionnaire can be completed easily in about 10 min. As it turned out, there were no major problems in processing the questionnaires. Based on the feedback from the doctors, several items on the item battery could be added to or modified regarding the perceived advantages and disadvantages of apps.

### Sampling

A total of 2138 completed questionnaires of the 2193 questionnaires processed were included in evaluation (32% response rate). The sample was structured as follows:

- Gender: 53% male, 47% female
- Average age: 54 (median: 55)
- Office setting: 46% in medium-sized and large towns or cities, 54% in small towns or rural areas
- Type of office: 51% individual doctor’s offices, 45% joint offices, 4% other
- Patients per quarter: 20% < 1000, 34% 1000–1500, 21% 1501–2000, 25% > 2000

### Ethics

During this study, no sensitive patient data was gathered or clinical tests performed. This is a strictly anonymized survey of a total of 2138 general practitioners. However, the authors of the study contacted the Ethics Commission of the State of Rhineland-Palatinate before beginning the study to ensure that it conformed with the medical professional code of conduct.

### Data analysis

SPSS 23.0 (IBM, Armonk, NY, USA) was used for data analysis. In addition to descriptive analysis an independent samples t‑test was used to detect significant differences between two groups. The significance level used was *p* < 0.001.

## Results

### Assessment and potential use of health apps

Of the respondents, 36% expressed a generally positive opinion towards health apps compared to 43% more sceptical respondents; 21% expressed no particular preference. Physicians in medium-sized and large towns and cities saw health apps in a considerably more favourable light than their counterparts in small towns and rural communities (44% vs. 27%, *p* < 0.001). Respondents below the average age saw these apps far more favourably than did their older colleagues (46% vs. 23%, *p* < 0.001). Assuming correct application, 39% thought that health apps would make a large or very large contribution to health promotion, whereas 50% expected this contribution to be rather low; 5% did not expect any contribution, and 6% did not respond.

The perceived benefits of apps varied by application area. Of the physicians asked, 90% considered the use of mHealth tools as beneficial in managing medicines and medical appointments such as vaccinations and preventive screening. In addition, 88% welcomed support from patient self-management in risk factors such as bodyweight, blood pressure, blood sugar and similar, and weight data such as steps taken, and fluid volumes ingested. A further 76% saw benefit in using apps with physical activities such as exercises followed by features helping patients maintain a healthy lifestyle such as diet management and smoking cessation (65%). Finally, 59% advocated app support in monitoring and treating chronic diseases.

Respondents associated health apps with both opportunities and threats (Table 1, total approval). Perceived benefits included patient motivation and compliance. However, many of the physicians feared increased workload with frequent app usage as patients perceived general practitioners as their contact person. Some of the doctors pointed to data privacy issues and fears of unwanted effects such incorrect measurements.

**Table 1:** Perceived opportunities and threats in health apps. Question: *Which of the following statements do you agree with? *(*N* = 2138)

Question: *Which of the following statements do you agree with? *(*N* = 2138)	Overall approval (%)
Health apps raise motivation and willingness amongst patients to do something for their health	60
Health apps mean more rather than less work for physicians due to the additional responsibilities they cause	55
Health apps are too complicated for many patients to use, which could result in false health data being collected and treatment failure in extreme cases	49
Health apps are often untrustworthy as they do not adequately ensure data privacy	44
Health apps improve patient compliance	41
Health apps detract from the personal element in doctor–patient relationships	40
Health apps generate a wash of data that hinder fast and effective patient treatment	36
Health apps provide support in briefing patients on health and disease issues	35
Health apps are too time-consuming for physicians and patients to use	31
Health apps encourage patients to self-diagnose and self-treat without seeking professional medical advice	27
Additional information covered by health apps help physicians treat patients more effectively and personally	25
Health apps make planning doctor’s appointments more effective	23
Health apps make consultation between physician and patient easier	21
Using health apps speeds up the process of identifying and diagnosing diseases and disease risks	21
Health apps relieve doctors and nurses as they no longer have to worry about the recording health data and measurements	15

### App use in patient care and physicians’ own experiences

Of the respondents, 61% estimated that up to 10% of their own patients used at least one app occasionally or frequently, 24% speculated on a usage frequency of 10–20%, and 8% assumed more than 20%.

Around one in four (24%) responded that they had many or at least some patients that had sent or brought their health data in printed or digital form—blood pressure or blood sugar diaries, asthma diary, stroke risk test and similar—to the doctor’s office (a few patients according to 54% and none according to 22%). A total of 29% of doctors in large cities responded that they had many or a moderate number of such patients compared to 15% of physicians in rural communities (*p* < 0.001).

One in four general practitioners (25%) indicated that they had frequently or occasionally had their own patients mention healthcare apps; 37% said that this had been rare, and 35% never. Physicians frequently or occasionally asked about health apps were above average in number in medium-sized to large urban areas and less frequent in small towns and rural communities (32% vs. 17%, *p* < 0.001).

Of all respondents, 18% had frequently or occasionally recommend specific apps to patients for prevention, lifestyle changes and/or treatment, whereas 26% had seldom recommended apps and 56% had never recommended them. These recommendations were given more frequently in urban doctor’s offices compared to rural settings (23% vs. 12%, *p* < 0.001). According to a response to an open-ended enquiry, the app recommendations most commonly given focused on electronic blood pressure or blood glucose journal apps and preventive apps encouraging exercise and fitness, weight loss and diet control. Lifestyle-supporting applications for diabetics and apps for stress management and increased resilience were also frequently recommended.

Physicians that recommended apps (*n* = 943) named a variety of criteria to be met before a health app could be considered for recommendation. These criteria mainly consisted of guarantees for data privacy (84%), ease of use, simplicity and usability (75%), personalisation options (55%) and a recognisable feature that would motivate patients towards health awareness and self-care in everyday life (such as gamification features as named by 52%). Some physicians (44%) placed importance on documented app benefits (e.g., certificates, studies, and reviews), and some (39%) saw compatibility with conventional doctor’s office software as important in allowing portability for health data.

Only a small number (23%) saw themselves as capable of distinguishing good from poor quality in apps, or claimed to have a good general overview of the apps available (15%). Only 22% saw themselves as capable of counselling patients on health apps. Developing on the above, physicians in urban environments saw themselves as capable more frequently than did physicians in small towns and rural communities (28% vs. 16%, *p* < 0.001). Urban general practitioners also researched health apps more frequently (50% vs. 34%, *p* < 0.001).

Later on in the survey, the respondents were asked to think about those amongst their patients that used health apps regardless of whether they were acting on doctor’s advice or not. Asked about their experience, 54% responded that health apps had generally shown a very favourable or favourable influence on preventive healthcare and/or recovery amongst their patients. Few at 15% saw a negative impact on patient wellbeing, while 14% did not know any patients that were using them; 17% did not respond to this question.

The question as to the legitimacy of general practitioners relying on data collected by patients using apps in treatment planning was met with controversy amongst the respondents. In all, 37% saw it is reasonable to include the data, but the majority at 57% were against it and 6% did not provide an answer. Finally, 44% of all the physicians up to the age of 54 agreed with the inclusion of app data in treatment planning compared to only 24% of the older physicians (*p* < 0.001).

Doctors with patients that had sent or brought health app data in printed or digital form to the doctor’s office (*n* = 1670) were asked whether they themselves had also used health app data in treatment planning: 54% stated they did, whereas 22% said they would never include app data.

### Approaches towards optimising the use of apps at doctor’s offices

The results from the preliminary study and literature research were used as a basis for various proposals towards optimising the use of apps in primary care for comparison (Table 2). Most of the respondents wanted to see authoritative data privacy and quality standards defined in order to make health apps more attractive in patient care. Some also expressed a desire for mandatory certification in new apps. Respondents saw importance in clarifying the legal issues involved in using apps in patient care as an urgent measure towards improving the general conditions for using these apps. Further system training programmes for physicians was also seen as important.

**Table 2:** Approaches to optimising health app integration

Question: *Out of the following suggestions on improving the quality of health apps, which do you think are especially important? *(*N* = 2138)	Overall approval (%)
Definition of authoritative data privacy standards for health apps to ensure the protection of consumers and patients	66
Legal definition of quality criteria that must be met by health apps to ensure their trustworthiness	54
Obligation of providers to have their new health apps certified before they reach the market	37
Audit by regulatory authority on each app before market launch	35
Question: *Out of the following suggestions on improving general conditions for the use of health apps, which do you think are especially important? *(*N* = 1070)	Overall approval (%)
The fee schedule should regulate payment for medical services in connection with apps (consultation service code for assessing and evaluating data documented electronically by the patient)	73
Physicians should not have to risk liability such as in medical malpractice suits arising from a faulty health app	68
Health app system training programmes for physicians, especially in primary care, with sufficient CME-certified training	54
Doctors should be able to select from a wide range of health or medical apps on prescription in treatment planning for patients	20
Insurance policy holders from all public health insurance organisations should receive bonuses or bonus programmes for using certain apps regularly and transferring the data to the health insurance	11

Following that, the physicians were asked whether they would be generally more likely to consider including health apps in patient care if the points they specified (Table 2) were implemented. 19% responded “Yes, far more likely” and 53% “Yes, somewhat more likely,” while 19% were against it, and 9% did not respond.

Referring to the possibility created by the federal government to prescribe health apps under certain circumstances, 47% of the respondents said they could imagine using this in the future; 24% said no; 26% were undecided; and 3% did not respond. Respondents saw potential application areas for the use of apps in prevention medicine such as exercise training and gymnastics, physical therapy and rehabilitation, and dietary control. Many also responded that the apps could also be used in diabetes as well as headache and backache.

## Discussion

### Main findings and interpretation

The survey encompassing 2138 primary care physicians in Baden-Württemberg demonstrates the ambivalent opinions towards health apps amongst general practitioners. Overall, there was a marked contrast between the perceived potential for motivation, patient briefing and training as well as compliance on the one hand, and concerns for data privacy, reliability, and usability on the other. Even so, most of the respondents reported positive observations on the use of health apps by their patients.

Younger general practitioners took a more open-minded approach towards health apps as has already been observed in other studies on the topic [21, 22, 24]. The same applies to the difference between urban and rural settings: The results clearly suggest that mostly urban general practitioners consider including apps in patient care as their patients are generally younger and more digitally aware [16, 30].

Most of the general practitioners were more restrained in actively raising the subject or recommending an app in patient consultation, a result that also tallies with those of preliminary studies [17, 18, 23]. It was more common for patients themselves to mention corresponding apps, this personal interest causing the physician to include the app in the care process. Some physicians would definitely be prepared to include app data in treatment planning for patients presenting to the office with this data [19]. However, most of the respondents would not feel capable of giving patients expert counselling on the apps available as most physicians found it difficult to gauge the trustworthiness and reliability of apps [20, 22]. In this context, the results of the study carried out are similar to a representative survey of doctors by the German health insurance company Barmer [31] from the summer of 2020. This showed that around half of the doctors rated health apps positively and would recommend or prescribe them in a patient interview. However, around 60% feel poorly prepared for advice on apps.

Most general practitioners expressed a desire for data privacy and quality standards in apps as well as clarification of legal issues involved in implementing the apps in patient care, a broad range of further training programmes and regulation on fee schedules corresponding to consultation and support services. Once all these conditions have been met, most of the respondents said they could imagine including health apps in patient care more intensively than before. Almost every other respondent responded positively to the opportunity provided by the German federal government to prescribe health apps.

The results from the survey indicate that general practitioners have recognised the potential of new mHealth apps, but this potential has not yet been fully utilised due to current reservations and uncertainties. In this respect, these results tally not only with the interviews from the preliminary study but also fit into the consensus from studies published up to now. However, it must be pointed out that so far there have been only a few studies that specifically deal with the use of health apps in the general practitioner setting. Other studies indicate that factors such as the lack of oversight and transparency have kept physicians from making thorough use of these apps, although physicians are aware of their potential benefits of apps in prevention and care [19, 21, 22, 24, 25]. There are parallels to the work of Byambasuren and colleagues [23], who showed that general practitioners are uncertain about the reliability of health apps. GPs also find it difficult to anticipate the ease of use of apps and the possibilities of integration into the patients’ everyday life. Given the ever-growing plethora of new product launches, not only citizens but also medical specialists are simply overwhelmed by the task of distinguishing between good and bad options [32]. Studies also show that many patients lose interest in using apps after a relatively short period of time. Rasche et al., for example, examined the prevalence of the use of health apps among older adults (60+) in Germany using an exploratory survey. It turned out that only around 17% of those surveyed use health apps on a permanent basis [33].

A metastudy by the Bertelsmann Foundation [7] concluded that the health system has not yet found a way of dealing with these new applications in a consistent fashion at system level. In the long term, health apps could offer great opportunities when it comes to dealing with problems such as a regional shortage of doctors and better regulating the lifestyle of high-risk patients [1]. However, this requires institutions that support both doctors and patients to obtain an overview of the range of apps and selecting reputable applications. In this context, not only the health insurance system and other health stakeholders could play an important role [34]—in Germany legislature has also reacted and created the conditions for apps to be systematically included in care in the future. It will be important to define a binding and clear catalogue of criteria under which conditions apps can be prescribed by doctors. The national health portal introduced in Germany based on the example of other European countries could also be helpful in the long term in promoting the use of high-quality health apps [38].

### Strengths and limitations

The survey was based on a qualitative preliminary study and therefore closely aligned to the perspective of a general practitioner. Apart from that, the survey also saw a relatively high response rate. Even so, the study cannot be regarded as representative due to the limited number of respondents and the emphasis on regional recruiting. The anonymization does not allow the tracing of how many doctors in the different regions of the state participated in the survey. Therefore, statements about the quality of the sample are only possible to a limited extent. In addition, the possibility cannot be excluded that physicians with a specific interest in the topic might have been especially heavily represented in the cohort. However, the broad spread of respondents regarding major characteristics is reflected in the sample taken.

Given the breadth and complexity of health apps as a topic, this exploratory study can only be viewed as an initial approach to the issue. Usage and efficacy studies remain as a research desideratum in the context of specific application and care situations defined by criteria such as patient characteristics, disease, stage of disease and aim of treatment. In this respect, it will not only be important to shed light on the perspective of doctors, but also to carry out patient studies using particular health apps. There is currently a lack of such studies. A study that examines the acceptance, current and potential use of apps in the family doctor setting provides a basis for decision-makers and healthcare providers to further expand the digital possibilities of healthcare and to develop strategies to motivate patients to use apps consistently. Initial studies already show findings on the influence of health apps on an improvement in prevention, precaution and compliance as well as on the promotion of a more health-conscious lifestyle [1, 4, 35].

## Conclusion

New opportunities in healthcare emerge as growing numbers of consumers use health apps [5, 7, 28]. Apps could be effective support tools for general practitioners to encourage and reinforce patients in their personal disease management, compliance, motivation, and healthy behaviour, while instilling preventive aspects of treatment [4]. The use of apps towards lifestyle improvement and treatment seems highly plausible in primary care.

As the results have shown, many general practitioners are aware of the potential that apps provide but still harbour a slew of critical concerns. For this reason, the possible added value that apps offer in healthcare cannot be fully utilised. These concerns need to be addressed for health apps to be used across the board in healthcare, specifically primary care.

The CHARISMHA metastudy [1] brought together a variety of recommendations for improved integration of health apps in healthcare. These recommendations included more intensive and thorough orientation amongst app manufacturers towards quality criteria such as ISO and DIN, administrative healthcare factors ensuring effective quality control, and defining reliable criteria regarding intended purpose and inclusion of standards pursuant to their categorisation as medical products [32]. The plans of the German federal government to categorise health apps as prescription healthcare products in the future may also lead to increased effort on the part of manufacturers in reaching quality standards, and this may in turn lead to a certain amount of disruption on the app market [1, 4, 12, 32].

In addition, clarifying the legal situation with regard to using health apps in patient care such as in liability for erroneous data measurement as well as defining an appropriate medical fee structure for medical services provided in connection with health apps will play a major role in the future use of health apps [16–18]. General practitioners also require across-the-board training programmes on the benefits and limitations of app use alongside potential strategies for regular digital tool integration to increase user acceptance among physicians and patients. Initial model projects and system reviews already exist [36, 37]. There also needs to be some entity to provide orientation in providing an overview of which apps are useful and trustworthy for the respective application; professional societies could fulfil this role. Finally, apps with proven benefits should be available on prescription with health insurance coverage; however, this would require greater statistical backing [1, 4, 32]. Health apps will only be able to develop their potential to the full as support tools in primary care once the necessary conditions have stabilised.
